# Risk of infection and conversion time from external to definitive fixation in open tibial fracture

**DOI:** 10.1186/s13018-024-05350-2

**Published:** 2024-12-23

**Authors:** Wazzan S. Aljuhani, Yasir A. Alshabi, Abdullah M. Alanazi, Meshal A. Alothri, Saleh A. Almutairi, Ziad A. Aljaafri, Abdullah M. Alzahrani

**Affiliations:** 1 Department of Orthopedic Surgery, Ministry of the National Guard – Health Affairs, Riyadh, Saudi Arabia; 2 King Abdullah International Medical Research Center, Riyadh, Saudi Arabia; 3 King Saud bin Abdulaziz University for Health Sciences, Riyadh, Saudi Arabia; 4 Department of Orthopedic Surgery, King Fahad Hospital, Ministry of Health, Madinah, Saudi Arabia; 5 Department of Orthopedic Surgery, Prince Sultan Military Medical City, Riyadh, Saudi Arabia

**Keywords:** Infection, Open fracture, Temporary external fixation, Tibial shaft fracture, Gustilo classification, Trauma

## Abstract

**Background:**

An open fracture of the tibia is one of the most common and dangerous type of open fractures. In the management of these injuries, the primary focus is on reducing the infection rate, as this is crucial for achieving the best clinical outcomes. This study aims to explore how provisional external fixation duration influences the rates of infection and union in open tibial shaft fractures.

**Methods:**

A retrospective study with a total of 55 patients who received temporary external fixation. Groups A (less than 12 days), B (12–24), C (25–36), and D (more than 36) were the four groups into which they were split according to the conversion time.

**Results:**

12.8%, 18.2%, 50%, and 100% of the infections were found in Groups A, B, C, and D, respectively. Significant (*P* < 0.05) differences were found throughout the four groups. The conversion time from external to definitive fixation was found to have a relationship with the occurrence of an infection (*P* = 0.004). A higher prevalence of infection was observed over time. However, no association was observed between infection and antibiotic duration or initial debridement time (*P* = 0.689 and *P* = 0.963, respectively).

**Conclusions:**

Results of this study demonstrate that the likelihood of infection increases when the change from external fixation to definitive internal fixation is delayed. Therefore, it is important to convert to definitive internal fixation immediately when the local and general conditions are favorable for doing so.

## Background

Open fractures are often associated with high-energy trauma, leading to frequent infections, and adversely affecting fracture healing [1]. Tibial shaft fractures occur more frequently than any other long-bone fracture, with high-energy motor vehicle accidents (MVA) causing 58% of all tibial fractures [2]. Due to the comparatively thin tissue covering of the tibial shaft, they are the most susceptible to open fractures of long bones [3]. Open fractures are ordinarily categorized into grades I, II, IIIA, IIIB, and IIIC as per the Gustilo-Anderson classification, which is prepared based on different characterization factors [4]. However, the incidence and distribution of open tibial shaft fractures in Saudi Arabia have not yet been reported. However, the last 10 years have witnessed an increase in the number of lower extremity fractures, reflecting an increase in the healthcare burden [5].

Open fractures are considered orthopedic emergencies and are managed with systemic antibiotics for the first 72 h after injury [6, 7], early irrigation and debridement, fracture stabilization, and soft tissue reconstruction. For definitive fracture stabilization, the use of intermedullary nail as a definitive internal fixator is the most optimal option for tibia shaft fracture due its high union rate [8]. However, provisional external fixation lowers the risk of infection in highly contaminated grade III fractures [9–11].

Since infection is one of the most frequent causes of nonunion, it is important to focus on prevention of the infection [12]. Prevention entails such measures as appropriate timing of debridement, wound closure, and timely change from temporary external fixation to definitive fixation [13, 14]. However, the best time to make this switch remains a matter of debate. If the original contamination is sufficiently handled and the soft tissue envelope is restored, definitive fixation can be carried out.

Several studies have established that infection rates range between 20 and 24% when the external fixator is eradicated at a mean of 51–69 days, with a 95% union rate after 1 year. On the contrary, however, a different study that removed the external fixator at a mean of 91 days showed a 10% infection rate, but after a year, it obtained a 100% union rate [13, 15, 16]. According to Bhandari et al., a shorter duration (< 28 days) of external fixation reduces overall infection risk; however, the union rate was 92% [17]. To the best of our knowledge, local data regarding external fixation duration and infection rate are limited. Therefore, this study explores how provisional external fixation duration influences the rates of infection and union in open tibial shaft fractures.

## Methods

This retrospective study was carried out at King Abdulaziz Medical City (KAMC), Riyadh, Saudi Arabia, to explore the relationship between infection rate and external fixation conversion time. The following were the inclusion criteria: age > 18 years, case with provisional external fixation before definitive fixation, and admission to KAMC between January 1, 2015, and January 1, 2021.

All patients included in the study were managed according to open fracture principles and damage control orthopedics. Antibiotics and tetanus immunoglobulin were initiated initially in the emergency department. Initial necessary measures for open wounds were taken prior proceeding to operating room for external fixation applications. After that, patients were followed until their conditions including but not limiting to soft tissue conditions, patients’ comorbidities, host variability and response to antibiotics were optimized and being ready for the definitive fixation.

The grouping of patients based on time taken for the conversion from external fixation to definitive fixation was based on the statistical analysis performed that showed significance, as there is no cut-off period presented in the literature.

There were 55 patients, who were grouped into 4 groups based on the time taken to make the switch from external to definitive fixation: Group A (< 12 days), Group B (12–24 days), Group C (25–36 days), and Group D (> 36 days). The Gustilo–Anderson classification was used for classifying open fractures and prophylactic antibiotics were administered as recommended.

The statistical analysis was conducted using IBM SPSS Statistics, Armonk, NY’s Statistical Package for Social Sciences version 25. The Student’s t-test investigated the association between numerical variables, while the chi-square or Fisher’s exact test investigated the relationship between categorical variables.

Consent was not required for this retrospective cohort study, and all data were kept within a secure place with access only given to the research team. No identification data were collected, privacy and confidentiality were assured.

## Results

Our study included 55 patients with a mean age of 36 (13.6) years. Most (49.11%) patients were overweight, followed by normal weight in 29.1%, then obesity in 16.4% of patients. The most frequent mechanism of injury was MVA (56.4%), followed by pedestrian-inflicted injuries (32.7%). And 10.8% of included patients sustaining injuries from another mechanisms. Most tibial shaft fractures were unstable in length (89.1%) and associated with fibular fractures (78.2%). The most common Gustilo–Anderson classification grade was II (*n* = 16, 29%), followed by I (*n* = 13, 23.6%), IIIB (*n* = 13, 23.6%), IIIA (*n* = 6, 10.9%), and IIIC (*n* = 6, 10.9%). The mean time to conversion from external fixation to definitive fixation was 15 days. The mean antibiotic duration and initial debridement period were 12.3 (8.4) days and 20.2 (16) hours, respectively (Table 1).

**Table 1:** Baseline characteristics

Variables		Mean (standard deviation)/*N* (%)
Age (year)		36 (13.6)
Body mass index	Underweight	3 (5.5)
	Normal	16 (29.1)
	Overweight	27 (49.11)
	Obese	9 (16.4)
Mode of injury	Motor vehicle accident	31 (56.4)
	Pedestrian inflicted	18 (32.7)
	Hit by a heavy object	2 (3.6)
	Explosive	2 (3.6)
	Motorbike accident	1 (1.8)
	Fall	1 (1.8)
Fracture stability	Length stable	6 (10.9)
	Length unstable	49 (89.1)
GCS		11.4 (4.4)
Associated fibula fracture	Yes	43 (78.2)
	No	12 (21.8)
Infection	Yes	11 (20)
	No	44 (80)

The Gustilo–Anderson classification was noted for 11 patients who developed infections: 7, grade IIIB; 2, grade II; 1, grade I; 1, IIIC grade (*P* = 0.002). Moreover, the conversion time from external to definitive fixation was directly related with the occurrence of an infection (*P* = 0.004). A higher prevalence of infection was observed over time. However, no association was observed between infection and antibiotic duration or initial debridement time (*P* = 0.689 and *P* = 0.963, receptively; Tables 2 and 3, and 4).

**Table 2:** Association between infection and the Gustilo–Anderson classification

Grade	Infection, N (%)		P value
	Yes	No	
I	1 (7.7)	12 (92.3)	0.024
II	2 (12.5)	14 (87.5)	
IIIA	0 (0)	6 (100)	
IIIB	7 (53.8)	6 (46.2)	
IIIC	1 (16.7)	5 (83.3)	

**Table 3:** Association between infection and the conversion time from external fixation to definitive fixation

Group	Infection, N (%)		P value
	Yes	No	
A	5 (12.8)	34 (87.2)	0.004
B	2 (18.2)	9 (81.8)	
C	1 (50)	1 (50)	
D	3 (100)	0 (0)	

**Table 4:** Association between infection and the initial debridement and antibiotic duration

Variables	Infection, Mean (standard deviation)		*P* value
	Yes	No	
Antibiotics (day)	13.2 (10)	12.1 (7.9)	0.689
Initial debridement (hour)	20.4 (17.9)	20.1 (18.8)	0.963

## Discussion

As can be seen above, the findings of this study suggest that the risk of infection is affected by the conversion time from provisional to definitive external fixation for open tibial shaft fractures. The longer the duration before conversion, the higher the risk of developing an infection. Surprisingly, no significant association was observed between the infection risk and antibiotic duration or initial debridement time. This suggests that factors other than the duration of antibiotic treatment and timing of initial debridement led to infection in open tibial fractures. Further investigation is warranted to explore these factors and their potential impacts on infection rates. Moreover, there is evidence that the higher the rate of having a Gustilo–Anderson classification grade, the higher the risk of developing an infection, owing to less soft tissue coverage and possibly poor blood supply [18]. In our study, grade IIIB patients had the highest incidence of infection followed by grade IIIC, grade II, and grade I. These results are similar to other studies which have demonstrated higher infection rates associated with higher Gustilo-Anderson grades. This is particularly true for IIIB and IIIC injuries owing to the large soft tissue injury [19, 20].

Trauma specialists have debated the timing between converting from the external fixator to a definitive fixation. Some studies have shown that a protocol of early delayed (approximately 2 weeks) definitive fixation has shown optimum results, as the risk of infection is remarkably low, especially for low-grade open tibial fractures [17, 19, 21]. In addition, it has been shown to decrease external fixation–related complications such as problems at the site of pins, nonunion, and malunion [21].

Studies have reported that prior to conversion to definitive fixation, pin tract–related infections must be excluded, as they have been shown to be a significant determining factor for developing infections. Therefore, after excluding pin site–related infections, a protocol compromising curettage, debridement, and irrigation of pin sites, as well as antibiotic coverage, minimizes the risk of infection [17, 22]. A great way to decrease pin site–related infections is by removing them as soon as possible and converting them to a definitive fixation. However, if an infection has developed, removing the pins and casting the limb are ideal until the infection has resolved and a definitive fixation can be performed [22].

Weaknesses of this paper include the relatively small sample size, which could lead the results being underpowered. Also, this is a single center study which could lead to a lack of generalizability of the findings. In other words, surgeons should aim to transition from temporary external to more permanent and internal fixation in a timely manner, as prolonged use of external fixation can cause more infections and other related health problems. The conversion should be performed immediately when the status of the patient and the injury allow.

## Conclusion

Our study provides valuable insights into the risk of infection and the conversion time from external to definitive fixation in open tibial fractures. Our findings highlight the significance of the Gustilo–Anderson classification in predicting infection risk and emphasize the importance of timely conversion to definitive fixation to reduce the likelihood of infection. Therefore, expediting the conversion to definite internal fixation must be attempted as soon as the local and overfall condition allows. Further research is required to provide a clearer understanding of the multifactorial nature of infection development in these fractures and identify additional strategies for infection prevention and management.

## Data Availability

No datasets were generated or analysed during the current study.

## Abbreviations

MVA: Motor vehicle accidents
KAMC: King Abdulaziz Medical City
BMI: Body mass index
