# Correction: Risk of infection and conversion time from external to definitive fixation in open tibial fracture

**DOI:** 10.1186/s13018-024-05440-1

**Published:** 2025-01-27

**Authors:** Wazzan S. Aljuhani, Yasir A. Alshabi, Abdullah M. Alanazi, Meshal A. Alothri, Saleh A. Almutairi, Ziad A. Aljaafri, Abdullah M Alzahrani

**Affiliations:** 1 Department of Orthopedic Surgery, Ministry of the National Guard –Health Affairs, Riyadh, Saudi Arabia; 2 King Abdullah International Medical Research Center, Riyadh, Saudi Arabia; 3 King Saud bin Abdulaziz University for Health Sciences, Riyadh, Saudi Arabia; 4 Department of Orthopedic Surgery, King Fahad Hospital, Ministry of Health, Madinah, Saudi Arabia; 5 Department of Orthopedic Surgery, Prince Sultan Military Medical City, Riyadh, Saudi Arabia

**Correction**: ***Journal of Orthopaedic Surgery and Research*(2024) 19:867**

10.1186/s13018-024-05350-2

In this article the author name Abdullah M Alzahrani was incorrectly written as Abdullah A. Alzahrani1.

The original article has been corrected.

